# Pre- and Posttreatment Findings of F-18 Fluorodeoxyglucose Positron Emission Tomography/Computed Tomography in a Case of Acute Q Fever

**DOI:** 10.4269/ajtmh.20-0012

**Published:** 2020-06

**Authors:** Yeon-Hee Han, Joo-Hee Hwang, Chang-Seop Lee

**Affiliations:** 1 Department of Nuclear Medicine, Jeonbuk National University Medical School, Jeonju, Republic of Korea;; 2 Cyclotron Research Center, Molecular Imaging and Therapeutic Medicine Research Center, Jeonbuk National University Medical School and Hospital, Jeonju, Republic of Korea;; 3 Department of Internal Medicine, Jeonbuk National University Medical School, Jeonju, Republic of Korea;; 4 Research Institute of Clinical Medicine of Jeonbuk National University-Biomedical Research Institute of Jeonbuk National University Hospital, Jeonju, Republic of Korea

**Figure 1.**
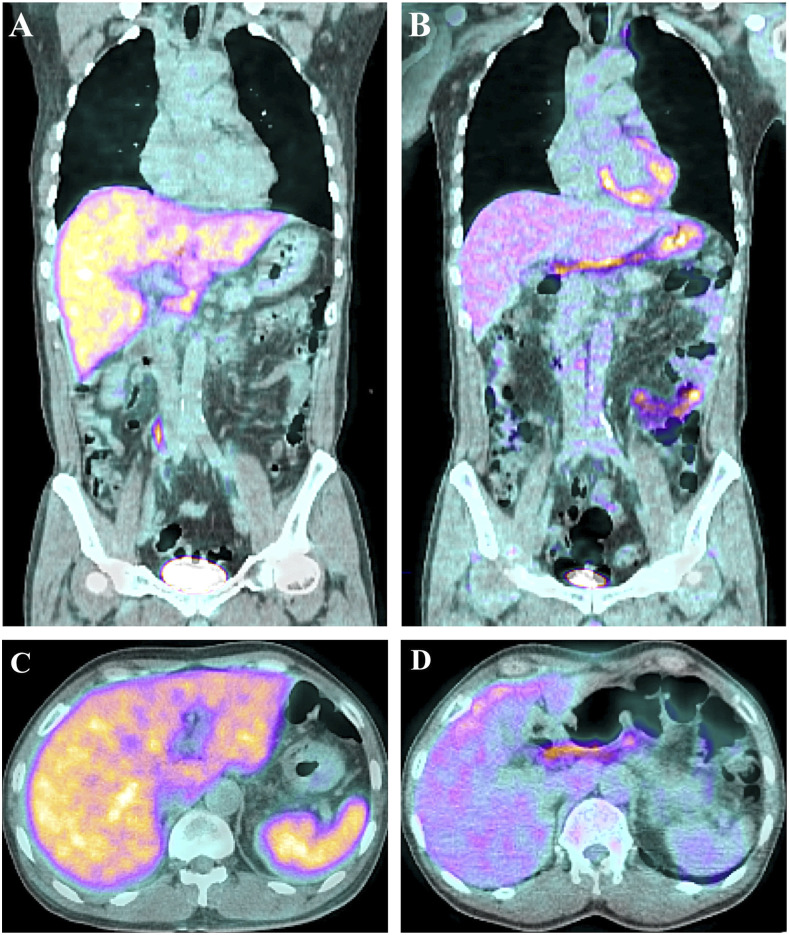
Coronal F-18 fluorodeoxyglucose positron emission tomography/computed tomography image before treatment (**A**) shows diffuse hypermetabolism in the liver, with hepatomegaly (AP diameter of the right hepatic lobe: 15.1 cm, SUVmax: 5.96, and SUVmean: 4.33). After treatment (**B**), the size and metabolism of the liver decreased to normal values (AP diameter of the right hepatic lobe: 13.7 cm, SUVmax: 2.53, and SUVmean: 1.77). An axial image before treatment (**C**) shows hypermetabolic splenomegaly (SUVmax: 5.59 and SUVmean: 4.46) that also normalized after treatment (SUVmax: 1.94 and SUVmean: 1.32) (**D**). Unlike for other infectious diseases, liver involvement was prominent. Hypermetabolic enlarged lymph nodes in the periportal and portocaval areas were also found on the initial image and returned to normal range after treatment. AP = anteroposterior; SUV = standardized uptake value. This figure appears in color at www.ajtmh.org.

After suffering from a fever for 1 week, a 31-year-old man was treated with antipyretics at a local clinic and was then admitted to an emergency room. Physical examination revealed mild icteric sclera and peritonsillar hypertrophy but no abdominal tenderness. The liver function test showed an elevated aspartate transaminase of 236 IU/L, alanine transaminase of 221 IU/L, total bilirubin of 2.70 mg/dL, and direct bilirubin of 1.99 mg/dL. His white blood count was 7,740/µL and C-reactive protein was 168.98 mg/L. As he had a persistently high fever despite ceftriaxone treatment, we performed F-18 fluorodeoxyglucose positron emission tomography/computed tomography (FDG PET/CT). Coronal and axial images before treatment (Figure 1A and C) showed diffuse hypermetabolism in the liver and spleen, with hepatosplenomegaly. The diagnosis was confirmed by a greater than 4-fold increase in indirect immunofluorescence assay IgG titer against *Coxiella burnetii* in phase II in paired serum samples collected 2 weeks apart. After oral doxycycline for 2 weeks, the patient’s symptoms improved. After treatment (Figure 1B and D), the size and metabolism of the liver and spleen decreased to normal values. Hypermetabolic enlarged lymph nodes in the periportal and portacaval areas on initial imaging also returned to a normal range after treatment.

Q fever is a globally important zoonotic disease caused by the bacterium *C. burnetii*, which can be recovered from goats, sheep, and cattle. Transmission occurs primarily through inhalation of aerosols from contaminated soil or animal waste.^1^ Because most patients with acute Q fever have nonspecific symptoms such as fever, chills, headache, anorexia, nausea, and myalgia, physicians often fail to suspect Q fever during the acute stage of the disease.

Isolated hepatitis presents from 49.2% to 100% more frequently than pneumonia in countries where the disease is endemic.^2^ F-18 fluorodeoxyglucose positron emission tomography/computed tomography is one of the most frequently used imaging modalities for cancer assessment; its clinical utility is widely spreading to other clinical fields such as infectious disease.^3^ F-18 fluorodeoxyglucose positron emission tomography/computed tomography has contributed to early detection and precise localization of infectious focus in patients with a fever of unknown origin, both of which are critical for prompt and appropriate treatment. Several reports have noted the clinical utility of F-18 FDG PET/CT in diagnosing Q fever through demonstration of endocarditis, osteoarticular localization, lymphadenitis, and vasculitis.^4^ Diffuse hypermetabolism in the liver and spleen with enlarged lymph nodes can be seen both in malignant conditions such as lymphoma, cholangiocarcinoma, and angiosarcoma and in infectious disease such as tuberculosis. The mechanism of F-18 FDG uptake in Q fever could be explained by the hematogenous spread of *C. burnetii* leading to infection of the liver, spleen, and lymph nodes, causing granulomatous lesions. In our report, F-18 FDG PET/CT showed specific findings of acute Q fever as intense hypermetabolism in the liver/spleen with hepatosplenomegaly. These findings provide key clues for early recognition of Q fever and for assessing the treatment response. A previous case report of acute Q fever hepatitis detected on FDG PET/CT image was very similar to the patient in this article.^5^ However, no posttreatment FDG PET/CT image was presented for that case. To our knowledge, our article is the first showing both pre- and posttreatment images of Q fever on F-18 FDG PET/CT.
